# Applications and Degradation of Proteins Used as Tissue Engineering Materials

**DOI:** 10.3390/ma2020613

**Published:** 2009-05-26

**Authors:** Hua-Jie Wang, Ling Di, Qiu-Shi Ren, Jin-Ye Wang

**Affiliations:** 1 Biomedical Engineering, School of Life Science and Biotechnology, Shanghai Jiao Tong University, 800 Dongchuan Road, Shanghai 200240, China; E-Mail: wanghuajie972001@sjtu.edu.cn (H.-J.W.); 2 Shanghai Institute of Organic Chemistry, Chinese Academy of Sciences, 345 Lingling Road, Shanghai 200032, China

**Keywords:** proteins, tissue engineering, biodegradation

## Abstract

This article provides an up-to-date review on the applications of natural polymers, i.e., proteins, as materials for tissue engineering. Proteins are one of the important candidates for tissue engineering materials based on their superior biocompatibility, biodegradation, bioresorbability, and so on. However, their inferior mechanical properties limit their broad application. Currently-available proteins for application in tissue engineering or drug delivery systems, such as fibrin, collagen, zein, silk fibroin, keratin, casein and albumin, and the biodegradation of tissue-engineered substitutes based on proteins are presented. Techniques of scaffold fabrication are also mentioned. Problems and future possibilities for development of protein-based tissue-engineered substitutes are also introduced in this review.

## 1. Introduction

Studies dating back to the 1990s demonstrated that tissue engineering is a promising alternative approach in treatment of damaged or lost tissues or organs, by overcoming the drawbacks in traditional tissue or organ transplantation, such as the insufficient number of donors, traumatic procedures and inflammatory reactions, etc. [1,2,3]. Proteins, as natural biodegradable materials and current progress in their applications in the tissue engineering, have generated considered attention, mainly due to the following reasons: (1) Proteins have excellent biocompatibility and biodegradability, especially because their degradation products, amino acids, are the basic components of life and can be resorbed as nutrients. Consequently, (2) some of them induce minimal tissue inflammatory responses. (3) Some proteins are available on a large scale, and there are low cost [4,5]. As a result, great effort has been expended in developing applications for proteins as tissue engineering materials [6,7,8,9].

Herein, we provide a summary of the applications of proteins as biomaterials, such as in tissue or organ substitutes containing bone and cartilage, skin and ligaments, or vascular, corneal and neural substitution, and as drug delivery carriers, starting with a description of the various types used, the fabrication techniques and degradation mechanisms, with particular attention to more recent developments in the field.

## 2. Types of Proteins Used as Biomaterials

### 2.1. Fibrin

Fibrin (also called factor Ia) is a fibrillar protein made from fibrinogen and involved in the clotting of blood. Based on these characteristics, fibrin monomers can be polymerized to form a 3-D mesh-shaped gel that is plastic, cohesible and degradable. This cross-linking process for 3-D fibrin scaffold fabrication is driven by activated factor III, which catalyzes the formation of covalent lysyl-glutamine bonds, rapidly linking γ-γ dimer [10,11]. Fibrin and its degradation products induce angiogenesis and promote cell attachment and proliferation. Therefore, fibrin has gained much attention in the fields of drug delivery and tissue engineering [12,13,14,15]. For example, Deutsch *et al.* used fibrin glue to coat ePTFE grafts and the grafts after being seeded with autologous endothelial cells were implanted in patients. The results demonstrated that *in vitro* endothelialization on fibrin glue surfaces could be accomplished and it promoted the beneficial clinical effects in elective infrainguinal bypass patients [16]. However, there are still some barriers to the further applications of fibrin. For example, the tissue engineering product derived from fibrin lacks strong mechanics because of the instability and solubility of fibrin over time *in vitro* and *in vivo* due to fibrinolysis. Therefore, fibrin always appears as coatings, films for cell adhesion, proliferation and differentiation, or as 3-D scaffolds for repair of cartilage and neuronal injury [11,17], but seldom for repair of sclerous tissues. The controllable degradation for fibrin products is also one of the major thrusts in application development [18].

### 2.2. Collagen and gelatin

Collagen is a long and fibrous structural protein that contains three peptide chains, which form a triple helical structure by intra-molecular hydrogen bonds between Gly and Hyp in adjacent chains [19]. Collagen is the main protein in the connective tissue in animals and the most abundant protein in mammals, accounting for about 25% to 35% of whole-body protein content [20]. *In vivo*, multiple collagen fibrils form into collagen fibers, which are a major component of the extracellular matrix and support most tissue and cell structures. Collagen has great tensile strength, and is the main component of fascia, cartilage, ligaments, tendons, bone and skin.

As a natural material, collagen has excellent biocompatibility, negligible immunogenicity, and high bio-absorbability. For example, Thumann *et al.* proved that collagen is non-toxic by morphology, viability and differentiation analysis. After 24 weeks of subconjunctival implantation, a collagen membrane didn’t show evidence of inflammation or fibrosis, and it was successfully absorbed in 17 weeks. The collagen membrane degraded slower in subretinal space and also didn’t elicit any rejection or inflammatory response [21]. Hong *et al.* prepared 2-D and 3-D matrices of type I collagen and found that the geometry and composition of matrices influenced the contextual activation of the ERK pathway, which resulted in different effects on cell phenotype [22]. Consequently, collagen has been widely used in tissue engineering and other biomedical applications, such as hemostatic agents [23,24]. For example, the importance of collagen in the extracellular matrix, and its role in the developmental cascade leading to new bone and cartilage from progenitors, implicates this molecule as a strong candidate material for a bio-mimetic approach to tissue engineering scaffold design [25]. Collagen has been widely employed in the construction of artificial skin substitutes used in the management of severe burns, and several commercial products from collagen have been marketed, such as collapat II^®^ (Biomet Inc.), Healos^®^ (Depoy Spine, Inc.), Collagraft^®^ (Nuecoll Inc., Zimmer Inc.) and Biostite^®^ (Vebas S.r.l.) [26].

If collagen is partially hydrolyzed under mild conditions, the three collagen strands separate into globular, random coils, producing gelatin. In contrast with collagen, gelatin has relatively lower antigenicity, while it still retains some of the information signals (such as Arg-Gly-Asp, RGD sequence) and may promote cell adhesion, differentiation and proliferation.

### 2.3. Zein

Zein is the major storage protein of corn and comprises 40-50% of total endosperm proteins. Zein belongs to the family of proteins known as prolamines due to their solubility in alcohol-water mixtures (60-95%), which is the major reason for their identification. This solubility property depends on its amino acid composition, which is characterized by an abundance of hydrophobic and uncharged amino acids, and includes such amino acids as leucine (20%), proline (10%), and alanine (10%). Zein is a heterogeneous mixture linked by disulfide bonds and has an average molecular weight of 44 KDa. The α-helical proportion of zein amounts to 50 – 60%, β-sheets comprise about 15%, and the remainder of the molecule is aperiodic [27]. Zein has been used widely as a coating agent in the pharmaceutical and food industries. In addition, zein has been applied as an adhesive, biodegradable plastic, chewing gum, fiber, cosmetic powder and inks [27,28]. In our group, we had proven that zein is a material that is biocompatible with the endothelial cells of human umbilical veins, human liver cells and mice fibroblast cells. Consequently, zein shows potential for application as a drug delivery carrier and as a scaffold for tissue-engineered bone/cartilage [29,30,31].

### 2.4. Silk fibroin

Silk fibroin is a natural fibrous protein produced by spiders or insects, such as *Nephila clavipes* and the domestic silk worm *Bombyx mori* [32]. Silk fibroin polymers consist of repetitive protein sequences with structural roles in cocoon formation, nest building, traps, web formation, safety lines and egg protection. Silk fibroin is currently being investigated for several biomedical applications due to its unique properties, such as its easy processing, impressive mechanical strength, environmental stability, biocompatibility and controllable proteolytic biodegradability, morphologic flexibility and because of the ability to undergo amino acid side chain modification to immobilize growth factors [33,34,35]. Tissue engineering scaffolds are the main product based on silk fibroin. Highly homogeneous and interconnected pores, controllable pore sizes (100 – 1,000 μm), suitable porosities (> 90%), degradability, better biocompatibility and useful mechanical properties (from several KPa to several MPa) are the characteristics of these scaffolds [36,37,38,39]. Recent progress on silk fibroin also demonstrated that a close similarity of nanometric silk fibroin scaffold to the natural extracellular matrix could induce the adhesion pathways of endothelial cells by up-regulating integrin-β1 expression compared with microfibrous samples, in addition, endothelial cells grown on nanofibrous silk fibroin scaffolds could recognize the nano-matrix as a contiguous substrate for growth through an integrin-dependent mechanism and formed a differentiated and interconnected cell layer [40]. In addition, silk fibroin has been used as a bioactive substrate delivery carrier because of its mild processing conditions. For instance, Wenk *et al.* fabricated salicylic acid and propranolol hydrochloride-loaded or insulin-like growth factor I-loaded silk fibroin spheres using laminar jet break-up. The encapsulation efficiencies for both bioactive substrates were close to 100%, and the release of insulin-like growth factor I occurred over seven weeks in bioactive form during a MG-63 cell proliferation study [41]. Additionally, films and scaffolds based on silk fibroin could be used to load the bioactive substrate; these loaded bioactive substrates still exhibited better bioactivity after processing. Examples of such films and scaffolds include silk fibroin film for the embedment of horseradish peroxidase, lysozyme and nerve growth factor [42,43], and electrospun silk fibroin scaffolds for embedment of BMP-2, IGF-I, alkaline phosphatase and staphylococcal protein A [44,45,46,47].

### 2.5. Keratin

Keratin is a tough, insoluble and structural protein that is a major component in skin, hair, nail, hooves and horns. Keratin molecules are fibrous, twisting around each other to form strands called intermediate filaments. Amino acid analysis of keratin showed its extraordinary high content of sulfur-containing amino acids, largely cysteine (7 – 20% of total amino acid residues), which forms inter- and intra-molecular disulfide bonds to provide the tissue with flexible and tenacious properties [48]. Keratin has good biocompatibility and been used in tissue engineering, owing to its cell adhesion sequences, such as arginine-glycine-aspartic acid (RGD) and leucine-aspartic acid-valine (LDV) [49,50]. For instance, keratin films prepared by the casting method or by the compression-molded method could support fibroblast cell attachment and proliferation [51,52]. Keratin hydrogel from human hair could enhance the activity of Schwann cells, increase their attachment and proliferation, and up-regulate expression of important genes [53]. In the mouse tibial nerve model, keratin hydrogel significantly improved electrophysiological recovery at an early time point of regeneration and produced better results in a long-term electrical and histological study [54]. In addition, a great amount of work has been done to fabricate the 3-D porous keratin scaffold by freeze-drying or compression-molding/particulate-leaching and proved that it is a bone substitute candidate [48,49,55].

### 2.6. Casein

Casein is a predominant phosphoprotein that accounts for nearly 80% of proteins in milk and cheese. Casein consists of a fairly high number of proline peptides and has no disulfide bonds. Therefore, there is relatively little secondary or tertiary structure. Casein is poorly soluble in water, exhibiting hydrophobic properties, and appears in milk as a suspension of particles. Casein is used in the manufacture of adhesives, binders, protective coatings, plastics (such as knife handles and knitting needles), fabrics and food additives [56,57]. As a tissue engineering material, casein is inexpensive, readily available, non-toxic and highly stable. Casein often appears as a drug delivery carrier, mostly in the form of microspheres. For example, Latha *et al.* have prepared theophylline-loaded casein microspheres using glutaraldehyde cross-linking, where the sizes are 710 – 850 μm in diameter and drug-loading capacity is approximately 54% [58]. Both the *in vitro* and *in vivo* release tests show good correlation, and could prolong drug release. In addition, casein also shows better loading properties for the lipophilic drug progesterone, where the microspheres were 75 – 200 μm in diameter, and the incorporation efficiency was about 61% [59].

### 2.7. Albumin

Albumin is a class simple, water-soluble and globular protein. They can be coagulated by heat and are found in egg white, blood serum, milk, and many other animal and plant tissues. Serum albumin is one of the most abundant proteins, comprising about 55% of blood plasma protein. The main applied form of albumin for biomaterials is as pharmaceutical microspheres, which are spherical, with a mean particle size ranging from nanometers to micrometers. In the mid-twentieth century, Rhodes and Zolle first prepared human serum albumin microspheres with mean sizes of 5 – 15 μm, which contained a γ radial source and were used for the determination of abnormal pulmonary circulation [60,61]. Up to now, albumin microspheres have been extensively investigated for drug targeting to various organs and tissues [62,63].

## 3. Applications of Protein Materials

Proteins are used as structural materials in Nature. For instance, keratin is used for thermal insulation in hair, collagen for mechanical support in connective tissues, and silk in spiderwebs. Their excellent characteristics have attracted the attention of researchers in the tissue engineering field. The roles of proteins in this field are mainly divided into two classes: the first is tissue engineering substitutes, which provide cell support for anchorage and adherence through specific cell-matrix interactions, and effectively guide cellular growth and development [57,64]; the second is bioactive substrates carriers, which could deliver the active molecules in time and space to the target sites, adjust the concentration-duration relationship, enhance stability and avoid side effects of the drugs [41,65,66].

### 3.1. Tissue engineering substitutes

Tissue engineering has emerged over the last several decades and together with regenerative medicine, this technology holds great promise for patients as a possible therapy for restoring damaged tissues and organ functions with the ambitious goal of avoiding organ transplantation. Tissue engineering substitutes are either cultured *in vitro* until functional enough for implantation or they are implanted right after cell seeding and allowed to develop *in situ* [67,68,69,70,71,72]. Therefore, the tissue engineering of functional tissues depends on the development of suitable scaffolds, which provide a physiological and deformable substrate for cell growth. As tissue engineering materials, proteins are attractive candidates based on their excellent characteristics.

#### 3.1.1. Tissue-engineered bone and cartilage

The first generation of bone or cartilage tissue engineering technologies is now available for clinical use. In fact, a series of standards have been established for ideal bone or cartilage substitutes, and up to now, efforts have been focused towards this aim [66]. Among the biomaterials applied, proteins are one of them and have the advantages of biocompatibility, biodegradability, and even bioactivity. In order to satisfy the design criteria for bone scaffolds, including high porosity, structural integrity and degradability at a rate commensurate with the elucidation of new extracellular matrix (ECM) by seeded cells. A number of methods have been developed for different proteins scaffold fabrication. Traditionally, there are templating and leaching method, freeze-drying method, chemical/enzymatic cross-linking method, 3-D printing method, solution evaporation method [64,70,73,74,75]. Recently, the techniques of controlling the scaffold architecture at nano-scale level have been paid much attention in tissue engineering: such as electrospinning, molecular self-assembly and phase separation.

#### Fibrin

An important characteristic of fibrin is its increasing instability and solubility over time *in vitro* and *in vivo*, due to fibrinolysis, such that fibrin scaffolds do not satisfy the demands of bone scaffolds for long-term stability, integrity and suitable mechanics properties. Fibrin is almost exclusively used as tissue-engineered cartilage [17]. The formation of the 3-D fibrin scaffold is nontoxic and occurs during the coagulation cascade when fibrinogen is cleaved by thrombin to form fibrin monomers, which then spontaneously polymerize to form a three-dimensional matrix [76]. It is known that the variation of fibrin parameters, such as fibrinogen concentration, thrombin concentration, ionic strength (such as Ca^2+^), and the use of aprotinin, can generate gels with different appearance, mechanical properties, and stability [16,17,77]. When fibrin is used in bone tissue engineering, it is always combined with inorganic materials, where these constructs exhibit superior mechanical strength to inorganic material alone [78]. For example, Osathanon *et al.* fabricated fibrin/calcium phosphate composite scaffolds with tightly controllable pore sizes, pore interconnection (50%), porosity (approximately 74%), and calcium phosphate deposition by sphere-templating and leaching fabrication methods [73]. Moreover, the animal tests indicated that these scaffolds could be degraded within 30 days of subcutaneous implantation and 45 days of calvarial defect implantation, and that these scaffolds promoted bone formation that was enhanced by one morphogenetic proteins-2 (BMP2).

#### Collagen

Collagen is a better natural biomaterial because of its negligible immunogenicity and excellent biocompatibility. Moreover, collagen is mechanically stable, and could be fabricated into 3-D scaffolds by chemical cross-linking techniques, forming a porous structure [79,80]. However, collagen scaffolds usually have insufficient mechanical properties, swell substantially in water and are vulnerable to enzymatic digestion. Therefore, collagen scaffolds usually act as tissue engineering cartilage [74]. Stark *et al.* tested the application of 3-D collagen matrices for cartilage tissue engineering. Reverse transcription-polymerase chain reaction (RT-PCR) and immunohistochemistry showed that the primary porcine chondrocytes differentiated and that the matrices promoted the formation of cartilage tissue [81]. Collagen has also been combined with other inorganic materials in order to match the mechanical demands of tissue-engineered bone [25,82,83]. For example, Pek *et al.* created a porous bioresorbable nanocomposite bone scaffold consisting of collagen and apatite that chemically, structurally and mechanically matched natural bone. In addition to excellent bioactivity for promoting MC3T3 cells attachment and proliferation, the highest compressive stiffness of the nanocomposite was to 37.3 ± 2.2 MPa and the yield strength was 2.7 ± 0.1 MPa, which matched best with trabecular bone [84]. Kim *et al.* developed a biomimetic nanocomposite of gelatin-HA (hydroxyapatite) via electrospinning, which significantly improved the osteoblastc cellular activity in comparison with pure gelatin [85].

#### Zein

For zein, we studied the feasibility of zein as tissue-engineered bone and fabricated 3-D porous scaffolds. Zein and its degraded product show good cell compatibility [29,30,31], and does not interrupt the adhesion, growth or proliferation of rat mesenchymal stem cells (MSCs); moreover, porous zein scaffolds have osteoconductive properties in the presence of dexamethasone [86]. The pore size of the scaffolds, which are interconnected by a number of smaller pores resulting from the salt leaching process, can be controlled. Moreover, the pore morphologies of porous zein scaffolds can be sphere-like or tube-like depending on the shape of porogen. Using proper fabrication technique, porous zein scaffolds also show good mechanical properties: the Young’s compression modulus ranged from 28.2 ± 6.7 to 86.6 ± 19.9 MPa and the compressive strength ranged from 2.5 ± 1.2 to 11.8 ± 1.7 MPa. This property could make the scaffolds able to sustain the forces from the surrounding tissues, and maintain structural support for cellular proliferation and bone matrix secretion to leave space for new bone tissue growth during scaffold degradation [87]. Furthermore, the brittleness of porous zein scaffolds can be improved by the addition of plasticizers. We also found that the maximum values of the compressive strength and modulus, the tensile strength and modulus, and the flexural strength and modulus reached 51.8 ± 8.7 and 563.9 ± 23.4 MPa; 3.9 ± 0.86 and 751.6 ± 58.85 MPa; and 17.7 ± 3.02 and 514.4 ± 19.02 MPa, respectively [86,88]. Electrospinning has been considered as a useful technique in scaffold fabrication to increase the tensile strength and mechanical property for special applications. Jiang *et al.* fabricated composite fibers with polycaprolactone (PCL)-core and zein-shell by coaxial electrospinning, which displayed improved mechanical properties in both elongation stress and the strain at break [89].

#### Silk fibroin

Silk fibroin can also be fabricated into 3-D structures, including hydrogels and sponges [33]. Silk fibroin hydrogels have been prepared from aqueous silk fibroin solution and are formed from β-sheet secondary structures. They are physically durable to swelling in aqueous solutions but do not dissolve. For example, Fini *et al.* injected silk fibroin hydrogels in femur defects of rabbits and observed greater trabecular bone volume and thickness, significantly higher mineral and rate of bone formation when compared to poly (lactide-glycolide) [90]. The pH values and Ca^2+^ concentration impacted the formation of silk fibroin hydrogels, and silk fibroin concentration and temperature could affect the pore size of hydrogels [33,91,92]. Silk fibroin sponges are characterized by more abundant pores than hydrogels, which could be fabricated by gas foaming or lyophilization. The porosity and pore size depend on the content and particle sizes of the porogens [93,94]. However, both silk fibroin hydrogels and sponges have a common characteristic: poor mechanical strength [36]. Consequently, they are frequently used as tissue engineering cartilage. For example, Wang *et al.* prepared 3-D porous silk scaffolds with pore sizes of 550 ± 30 μm by the aqueous process. According to the results of confocal microscopy, real-time RT-PCR, histology and immunohistochemistry, they concluded that MSCs and chondrocytes could adhere, proliferate and differentiate along the chondrogenic lineage in the scaffolds. The rather homogeneous cell and ECM distribution was due to the unique features of the scaffolds, including rough and hydrophilic surfaces, and excellent pore interconnectivity [95,96].

#### Keratin

Keratin has very high potential as a material for scaffolds for tissue engineering, but up to now, all fabricated keratin scaffolds have been sponge-like. That is to say, these keratin scaffolds have good flexibility and poor strength but they are still largely utilized as cartilage substitutes. The fabrication techniques for keratin scaffolds include lyophilization, compression-molding/particulate- leaching, and the combination of both of them [48,49,55]. These techniques could yield the desired microstructures, such as the regulated pore sizes, high porosity and interconnected pore network. These 3-D characteristics, biocompatibility, degradability, and mechanical properties are what make 3-D keratin sponges good tissue-engineered cartilage candidates [50,97].

#### Casein and albumin

There are only a few reports on the use of casein and albumin as tissue engineered bone or cartilage, and they were fabricated as composites with inorganic materials. Ritzoulis *et al.* have prepared porous caseinate-hydroxyapatite composite for bone tissue engineering, but caseinate was used only to form caseinate-stabilized olive oil-in-water emulsions and made the hydroxyapatite link to the oil droplet surface by caseinate-calcium phosphate interactions [98].

#### 3.1.2. Tissue-engineered skin

Tissue engineered skin has been used clinically for 25 years and has developed greatly during this time [99]. Various skin substitutes have been developed in order to treat acute and chronic skin defects. Besides organic synthesized materials, such as poly-L-lactide (PLLA), PCL and polyglycolic acid (PGA), proteins are among the most successful materials applied as skin grafts [100]. For example, fibrin is a good skin substitute; fibrin gel could be produced from the patients’ own blood and used as an autologous scaffold for the seeded fibroblasts without the potential risk of a foreign body reaction. Krasna *et al.* prepared a fibrin-based skin substitute from commercial fibrin glue, and proved that it could enhance the colony-forming efficiency of keratinocytes, enable the cultivation of cells at low seeding densities, and could be safely used to treat a number of skin defects [101]. Wu *et al.* performed skin implantation in the rat model using fibrin as the skin substitute, and found that the fibrin interface was completely replaced with fibrovascular tissue by postoperative day 10 [102]. Additionally, collagen is also a very successful skin substitute material in the clinic and among FDA-approved products. Integra and Apligraf have found their way into the clinical treatment of burn victims and other patients afflicted with skin disorders that require skin graft treatment [103,104,105]. King *et al*. had investigated the biocompatibility of Integra in the clean surgical wounds of 20 guinea pigs and proved that Integra is readily biologically incorporated into surrounding viable tissue [106]. Baker and Iorio effectively reversed the pathology of a chronic wound of type II diabetes by the application of an Apligraf skin graft substitute along with autologous platelet-derived growth factor [107]. Nanotechnology has also been applied in this field. Coating electrospun collagen with PCL, Venugopal *et al.* fabricated a scaffold for dermal tissue engineering with mechanical properties similar to skin and with the ability to support the attachment and proliferation of human dermal fibroblasts [108].

#### 3.1.3. Tissue-engineered ligament

Ligaments are bundles of fibrous connective tissue that facilitate the stability and movement of normal joints and are constantly subjected to repeated motion and occasionally also to high physiological loads. Therefore, the ideal tissue-engineered ligament should have sufficient tensile strength and stress-strain behavior, high porosity, be biodegradable at a suitable rate and cause a minimal inflammatory response *in vivo*. Collagen is one viable candidate in this regard because of its excellent biocompatibility-like enhancement of cell attachment, proliferation, and the production of ECM. However, collagen scaffolds lack structural reinforcements, and the parallel arrangement of collagen fibers in scaffolds aligned with the direction of stress may cause long-term failure due to fatigue, creep, and abrasive wear [109]. Recently, research on silk on tissue engineering ligament exhibits a better solution. Scaffolds based on silk fibroin have unique mechanical properties in fiber form, excellent biocompatibility, cell-controlled degradability, and versatile processability. For example, they have a maximum load of about 2,337 N, an elastic modulus of about 354 N/mm and a strain at failure of about 38%, which is similar to native ligament. Consequently, silk is a potential candidate scaffold material for tissue-engineered ligaments when used alone or combined with other materials [110,111,112,113].

#### 3.1.4. Other tissue-engineered substitutes

In addition to contributing to engineered tissue substitutes for skin, bone and cartilage, and ligaments, proteins show the most exciting potential as other tissue-engineered substitutes with the development of scaffold fabrication technologies and scaffold materials such as fibrin scaffolds and collagen films, for vascular substitution and neural substitution [16,114,115,116,117,118], and silk fibroin film for tissue engineering cornea [32]. Very recently, the electrospun collagen scaffolds showed good biocompatibility with vascular cells under physiologic conditions and resisted adherence of platelets when exposed to blood. When implanted *in vivo*, these scaffolds retained their structural integrity over 1 month [119]. The electrospun silk fibroin/gelatin blend nanofibers showed excellent mechanical properties and good biocompatibility with human umbilical vein endothelium cells and mouse fibroblasts, which would be a good implant for blood vessel engineering application [120].

### 3.2. Drug delivery carriers

The tissue engineering strategies include repairing the defective or lost tissue with manufacturing-based constructs [121]. Successful tissue regeneration cannot always be achieved by the combination scaffolds and cells alone. Therefore, in addition to scaffolds and cells, signaling molecules could endow the scaffolds with activity to direct cellular function in the tissue repair processes such as cell migration, differentiation, proliferation and organization in a functional tissue. However, these signaling molecules are not simply mixed with or absorbed onto the scaffolds, but can be controllably released [122,123,124]. Controllable release means to the necessary site, at a suitable concentration, within the therapeutic range, and in the suitable time period, in order to direct the living body system towards the process of tissue regeneration [125]. Therefore, carrier materials could adjust the concentration-duration relationship of signaling molecules, that is to say, the expected effects can be maximized by localizing the signaling molecules at the site of action, while the chronic and systemic side effects can eventually be minimized. Then, carrier materials provide signaling molecules with protection from *in vivo* degradation and prolong their half-life in the body, thus extending the duration of scaffold bioactivation and allowing these factors to be released at preprogrammed rates [121].

#### 3.2.1. Collagen

The tissue-engineered constituents based on collagen as signaling molecules carriers have been tested quite extensively. Ueda *et al.* fabricated collagen sponges using the thermal cross-linking method [126]. Transforming growth factor β1 (TGF-β1) loading is accomplished by the swelling of collagen sponges, and the hydrophobic interaction between TGF-β1 and collagen is suggested to be the main force for the immobilization of TGF-β1. The release of TGF-β1 depends on the enzymatic degradation of collagen sponges according to good agreement of TGF-β1 retention with the amount of collagen sponge remaining *in vivo*. What is more important is that the TGF-β1-loaded collagen sponges enhanced the bone mineral density in the bone defect and promoted the bone repairing compared with empty collagen sponges, demonstrating the high biological activity of TGF-β1. In addition, collagen sponges also show a better loading effect on BMPs for bone regeneration [127,128], on nerve growth factor (NGF) for neuronal differentiation [129], and on vascular endothelial growth factor (VEGF) for enhancement of vascularization within engineered tissues [130,131].

#### 3.2.2. Silk fibroin

Similarly, loading the signaling molecules in scaffolds for the development of silk fibroin as a tissue-engineered material is necessary in order to endow them biological activity. For instance, Kirker-Head *et al.* studied the effect of BMP-silk composite matrices on critically-sized femoral defects, and the results implied its clinical potential as osteopromotive implants [132]. Uebersax *et al.* studied silk fibroin scaffold as an insulin-like growth factor I (IGF-I) carrier to induce chondrogenic differentiation of human MSCs, and found that the IGF-I release kinetics were impacted by the manufacturing parameters of scaffolds, including pH, methanol treatment and drug loading [45]. They also studied silk fibroin matrices as NGF carriers to support the proliferation and maturation of nerve cells, and the results demonstrated the potential of silk fibroin-NGF in peripheral nerve repair [43].

#### 3.2.3. Zein

In a previous study, we tried to load signaling molecules using zein as the carrier, and found that zein could be fabricated into microspheres by a phase separation method based on its solubility, and show better loading function for hydrophobic/hydrophilic molecules [30,31]. However, the encapsulation efficiency for both types of molecules is different, and the former (e.g., ivermectin is 68.51 ± 0.48%) is higher than the latter (e.g., heparin is 20.4 ± 2.6%). The encapsulation efficiencies of ivermectin and heparin varied with the ratio of zein to both of drugs and the sizes of the microspheres could be controlled by the zein concentrations. Furthermore, we fabricated a 2-D film using heparin-loaded zein microspheres and controlled its drug-content and drug-release by changing the drug loading efficiency of the microspheres and the thickness of the film. In addition to the typical biphasic release characteristic, characterized by an initial “burst effect” (phase 1) followed with controlled- and sustained-release (phase 2), the drug in the film could not be released completely because of aggregation among zein microspheres, and the drug release exhibited “non-chemical denudation and incompletely release”.

#### 3.2.4. Fibrin, gelatin and keratin

There are a limited number of papers describing the use of fibrin, gelatin or keratin as signaling molecule carriers in tissue engineering, but these still illustrate the potential in this field [133,134]. For example, Willerth *et al.* combined fibrin scaffolds, embryonic stem cells, and growth factors, including neurotrophin-3 (NT-3), sonic hedgehog (SHH), and platelet-derived growth factor (PDGF), by an affinity-based method [135,136,137]. The ratio of the affinity reagent to growth factor could affect the release and retention period of growth factor in fibrin scaffolds, and optimal growth factor doses in the scaffolds could be obtained, which had significant effects on the viability and differentiation of embryonic stem cell-derived neural lineage precursor cells. Ehrbar *et al.* created a fibrin film conjugating VEGF and demonstrated that this film could prolong VEGF release and significantly enhance vascularization [138]. In addition, Patel *et al.* prepared BMP2-loaded gelatin microspheres and fabricated composite scaffolds using gelatin microspheres and poly (propylene fumarate). The *in vivo* results showed that the release of BMP2 from the composite scaffolds exhibited minimal burst and linear release up to 28 days [139]. In summary, combining the scaffold with a suitable growth factor and cytokine is one of the practical possibilities for further enhancement of tissue repair.

## 4. Degradation of Protein Biomaterials

Ideally, for clinical use in bone regeneration, scaffolds should gradually degrade and be fully replaced by natural bone tissue [79]. Although there has been a great deal of work on the degradation of three-dimensional scaffolds, studies on protein scaffolds are still deficient and not systematic. The limited studies that exist demonstrated that factors influencing the degradation rate of protein scaffolds are highly dependent on protein type and physical and structural properties of scaffolds, such as scaffold shape, size, pore interconnectivity, porosity and surface morphology [140,141,142,143]. In addition, environmental factors, such as the addition of inhibitors or promoters to proteases, cross-linking between protein molecules, loading conditions and biocompatibility, also play a role in the degradation of protein scaffolds *in vivo* [144,145]. The degradation times for various scaffolds can range from weeks to years.

### 4.1. Protein type

Protein type can influence the degradation rate of scaffolds due to the specificity of relevant enzymes. For example, van Amerongen *et al.* fabricated 3-D scaffolds, based on collagen, for the replacement of injured myocardium. The results showed a high number of neutrophils and high expression of active matrix metalloproteinases (MMPs) around the scaffolds after 14 days of implantation. They also considered that the scaffolds had been enzymatically degraded by high levels of active MMPs secreted by neutrophils. The reasons for this lay in the following facts: (1) the neutrophils clustering around the collagen scaffolds express both MMP-8 and collagenase activity at the same time, (2) MMP-8 expression coincides with the scaffold degradation, and (3) MMP-8 is known to cleave type I collagen [140]. However, in our previous study, we found that collagenase had a weaker effect on zein scaffolds *in vitro*, and that the degradation of porous zein scaffolds *in vivo* could be maintained for eight months [31,88]. Similarly, collagenase could degrade silk fibroin scaffolds, but the effect is weaker [141]. Therefore, scaffolds based on silk fibroin might remain *in vivo* for a longer time [39].

### 4.2. Physical properties of scaffolds

Enzymatic degradation proceeds from the surface of the protein scaffolds, in a process called “surface erosion”. Therefore, the physical properties, including shape, size, porosity, pore size, pore-interconnectivity and surface morphology of the scaffold, are the important factors that control the degradation rate [39,141]. Generally speaking, scaffolds with a smaller size, higher porosity, and smaller diameter of pores are more preferable for providing a larger surface per volume ratio, and consequently provide more action on the site of proteases. Therefore, the degradation of these scaffolds is faster. We demonstrated this point using porous zein scaffolds with the different factors mentioned above, and the results showed that pore size had a weaker effect than other factors on the degradation rate *in vitro* [86]. However, the *in vivo* studies exhibited the important effect of pore size on scaffolds degradation, which is due to enhancement of the functions of specific cells by specific pore sizes, consequently leading to more protease secretion. For example, although there is hardly any consensus regarding the optimal pore size for effective in-growth of bone, the reported sizes range from 75 μm to 600 μm, depending on the different materials [142]. On the other hand, for in-growth of fibro-cartilaginous tissue, the recommended pore size ranges from 200 to 300 μm [143]. In view of this, the degradation rate of the protein scaffolds could also be influenced by the factors affecting cell functions, such as pore-interconnectivity and surface morphology. For example, control of surface topography and roughness is important to allow the migration of cells on the scaffolds’ surface. The rougher surface also enhances the diffusion rates to and from the scaffolds and facilitates vascularization, and improves the supply of oxygen and nutrients as well as waste removal.

### 4.3. Other factors

In addition to the aforementioned factors, there are other factors affecting the degradation of protein scaffolds, such as inhibitors or promoters to proteases, the cross-linking between protein molecules, processing conditions and the biocompatibility of scaffolds. For instance, in order to extend the longevity of fibrin *in vivo*, one approach is to use fibrinolysis inhibitors, primarily protease inhibitors such as aprotinin or tranexamic acid that are added to the fibrin gel and/or as a supplement to the cell culture medium, and these help slow degradation, thereby partially stabilizing the fibrin gel shape [5,6,8]. Additionally, variation of gelation parameters such as the concentration of fibrinogen and thrombin, and ionic strength, enormously influence gel appearance, mechanical properties, and stability. Eyrich *et al.* found that a final fibrinogen concentration of 25 mg/mL or a higher Ca^2+^ concentration of 20 mM and a pH between 6.8 and 9 led to gels that were transparent and stable for three weeks, the duration of the experiment. However, when these parameters were out of these ranges, the gels could be dissolved faster [17]. Wang *et al.* proved fabricated the aqueous-derived silk fibroin scaffolds and HFIP-derived silk fibroin scaffolds, and found that host immune system responses have significant impact on the *in vivo* behavior of both silk fibroin scaffolds [39]. The worse the compatibility of the scaffolds, the bigger the number of fibroblasts that appear around the scaffold. Consequently, the thicker capsule will prevent the infiltration of cells and the degradation of the scaffold is slower. Among the cells, neutrophils can secrete active MMPs, which enzymatically degrade the protein scaffolds. Macrophages also play an important role during host immune system responses to protein scaffolds. However, they take part in the rapid clearance of apoptotic neutrophils during inflammation, preventing a prolonged inflammatory response caused by the release of toxic intracellular contents associated with secondary necrosis [140,144,145].

In summary, an important protein characteristic is an increasing instability and solubility over time *in vitro* and *in vivo*, due to enzymolysis. Long-term stability and mechanical integrity are essential for cells that require sufficient time and stiffness to produce their tissue-specific matrix. Therefore, adjustment of above factors could control the degradation of the protein scaffolds.

## 5. Summary and Future Outlook

In summary, tissue engineering is one of the most exciting interdisciplinary and multidisciplinary research areas today, and is growing exponentially. Protein is an important candidate for tissue engineering because of its excellent biocompatibility, biodegradability, and even bioactivity. Protein scaffold materials and fabrication technologies play a pivotal role in tissue engineering, and are fast evolving. However, the problems with existing protein materials can be summarized as follows:

(1) Safety – The risk of disease transmission should be eliminated or reduced, where the use of recombinant protein may be an option to do this;
(2) Mechanics – A major issue hindering the widespread use of proteins as tissue engineering materials has been the limited mechanical strength they possess. Improvement of the mechanical properties is still a challenge for tissue engineering substitutes based on proteins, especially when they are used as sclerous tissue substitutes. Both protein modification and combination with other materials are promising ways to address this;
(3) Vascularization – The poor angiogenesis of substitutes *in vivo* is one of the major obstacles to the successful application of tissue engineering substitutes, and protein materials are not the exception. The addition of active molecules in protein-based tissue engineered substitutes will be a possible way to solve this problem. In addition, the use of co-culture systems consisting of different vascular cells to construct the vascularized tissue engineered substitutes is also a promising way [70].
